# Different Molecular Forms of TFF3 in the Human Respiratory Tract: Heterodimerization with IgG Fc Binding Protein (FCGBP) and Proteolytic Cleavage in Bronchial Secretions

**DOI:** 10.3390/ijms232315359

**Published:** 2022-12-06

**Authors:** Jens Weste, Till Houben, Sönke Harder, Hartmut Schlüter, Eva Lücke, Jens Schreiber, Werner Hoffmann

**Affiliations:** 1 Institute of Molecular Biology and Medicinal Chemistry, Otto-von-Guericke University Magdeburg, 39120 Magdeburg, Germany; 2 Section Mass Spectrometry and Proteomics, Diagnostic Center, University Medical Center Hamburg-Eppendorf, 20246 Hamburg, Germany; 3 Department of Pneumology, Otto-von-Guericke University Magdeburg, 39120 Magdeburg, Germany

**Keywords:** lung, TFF3, trefoil factor, IgG Fc binding protein, FCGBP, innate immunity, BAL, neutrophil elastase, von Willebrand factor, DMBT1

## Abstract

The polypeptide TFF3 belongs to the trefoil factor family (TFF) of lectins. TFF3 is typically secreted from mucous epithelia together with mucins. Both intestinal and salivary TFF3 mainly exist as disulfide-linked heterodimers with IgG Fc binding protein (FCGBP). Here, we investigated bronchial tissue specimens, bronchial secretions, and bronchoalveolar lavage (BAL) fluid from patients with a chronic obstructive pulmonary disease (COPD) background by fast protein liquid chromatography and proteomics. For the first time, we identified different molecular forms of TFF3 in the lung. The high-molecular mass form represents TFF3-FCGBP oligomers, whereas the low-molecular mass forms are homodimeric and monomeric TFF3 with possibly anti-apoptotic activities. In addition, disulfide-linked TFF3 heterodimers with an M_r_ of about 60k and 30k were detected in both bronchial secretions and BAL fluid. In these liquids, TFF3 is partly *N*-terminally truncated probably by neutrophil elastase cleavage. TFF3-FCGBP is likely involved in the mucosal innate immune defense against microbial infections. We discuss a hypothetical model how TFF3 might control FCGBP oligomerization. Furthermore, we did not find indications for interactions of TFF3-FCGBP with DMBT1gp^340^ or the mucin MUC5AC, glycoproteins involved in mucosal innate immunity. Surprisingly, bronchial MUC5AC appeared to be degraded when compared with gastric MUC5AC.

## 1. Introduction

Human TFF3 (formerly termed hP1.B) is a secretory polypeptide consisting of 59 amino acid residues [1], which belongs to the trefoil factor family (TFF) of lectins (for reviews, see [2,3,4]). TFF3 predominantly undergoes exocrine secretion by most mucous epithelia, such as the intestine, lung, salivary glands, uterus, and vagina [1,3,5,6,7,8,9]. Here, TFF3 is secreted together with mucins [10]. Furthermore, TFF3 is also secreted in an endocrine manner, e.g., by the hypothalamus [3,11]. TFF3 is the predominant TFF peptide in the human respiratory tract, where it is mainly synthesized by mucous acini of submucosal glands (together with the mucin MUC5B) and in varying amounts by surface goblet cells (mainly together with the mucin MUC5AC) [5]. This has been confirmed by transcriptional analysis at single-cell resolution [12]. TFF3 (together with TFF1) was also detected in the epithelium and submucosal glands of the human nasal mucosa [13] as well as in nasal polyps [14]. Of special note, the situation in the murine respiratory tract is very different, where TFF2 is the predominant TFF peptide and TFF3 is not detectable [15]. Under pathological conditions, TFF3 is increased in bronchoalveolar lavage (BAL) fluid from patients with chronic obstructive pulmonary disease (COPD) [16,17] as well as in the serum and sputum of COPD and asthma patients [18]. Furthermore, in cystic fibrosis (CF) airways, TFF3 synthesis increased in surface goblet cells, whereas the elevated expression of TFF1 and TFF2 occurred in mucous cells of submucosal glands [12].

Thus far, the function of TFF3 has not been elucidated unambiguously. In contrast to previous suggestions, its motogenic and anti-apoptotic effects are rather weak (concentration range of about 10^−6^ to 10^−7^ M; reviews: [3,4]). Of note, TFF3 has been reported to play a role for the viscioelastic properties of the cervical mucus plug [19]. This might be related to a weak lectin activity allowing binding, e.g., to a *Helicobacter pylori* lipopolysaccharide [20] and probably a GlcNAcα1→4Gal→R moiety [4,21]. Furthermore, there are indications that TFF3 promotes human airway epithelial ciliated cell differentiation [22,23]. This could be due to a lectin-triggered receptor activation/blocking by TFF3 [4,24].

As a hallmark, TFF3 contains an odd number of cysteine residues, i.e., seven, which is unusual for secretory proteins. As the oxidation machinery of the endoplasmic reticulum enforces disulfide bond formation in secretory proteins [25], TFF3 is expected to occur mainly as a disulfide-linked homodimer or a heterodimer. Exposed free thiols act as intracellular retention signals for unassembled secretory proteins, which are then subject to degradation [26]. In line with this, both human intestinal and salivary TFF3 mainly exist as disulfide-linked heterodimers with IgG Fc binding protein (FCGBP) [27,28,29].

FCGBP is an unusually long cysteine-rich repetitive glycoprotein [30] of most mucous epithelia and their glands, such as the alimentary, respiratory and urogenitary tracts and also their fluids, where it is co-expressed with TFF3 [8,9,30,31]. The repetitive units share similarity with the D assembly domains of von Willebrand factor (vWF), which alters its shape in a shear-dependent manner [32], as well as gel-forming mucins from frogs to human [33,34,35]. In the human and murine stomach, FCGBP even forms heterodimers with TFF1 [36,37], the latter having high similarity with TFF3 [3,4]. FCGBP is ubiquitous in vertebrates [38] and probably has a general function for the mucosal innate immune defense together with mucins [28,29,39,40]. For example, FCGBP is a highly up-regulated early defense gene in catfish skin after bacterial infection [41]. Furthermore, certain disulfide bridges of FCGBP might be sensitive to shear forces [42], which could be of particular importance for the viscoelastic properties of the respiratory tract mucus. Currently, there are no indications that FCGBP interacts with mucins [42].

The respiratory mucus mainly consists of linear bundles of MUC5B polymers (diameter of about 25 µm) [35,43]. These bundles are mainly formed from submucosal glands, and to a lesser amount from surface goblet cells [12]. They efficiently clean the tracheobronchial surface and remove bacteria. In contrast, MUC5AC is released exclusively from surface goblet cells; it forms tightly organized networks with a high degree of branching and is stiffer and more viscoelastic when compared with MUC5B [43]. It is assumed that MUC5AC sticks more to the surface epithelium and also coats the MUCB mucin bundles [43,44]. The ratio of MUC5AC/MUC5B is about 0.1 in sputum [45] and 0.3 in BAL fluid, respectively [17]. As particularly MUC5AC expression is strongly up-regulated by immune reactions of both the Th1 and Th2 type, it is the major cause of problems in obstructive lung diseases [35,43]. For example, the MUC5AC/MUC5B ratio is raised to 1.5 in the BAL fluid of asymptomatic smokers [17] and 0.4 in the sputum of CF patients [45].

Based on our previous studies on human intestinal and salivary TFF3 [27,28], we characterized here for the first time the molecular forms of TFF3 in bronchial tissue specimens, bronchial secretions, and BAL fluid by the help of size exclusion chromatography (SEC). The patients had a COPD background. Besides heterodimeric TFF3-FCGBP, we identified homodimeric and surprisingly also monomeric TFF3 with an unusual unpaired cysteine residue. The low-molecular-mass forms could exert an anti-apoptotic effect and a protective function against damage by reactive oxygen species (ROS), respectively. Furthermore, we detected novel disulfide-linked TFF3 heterodimers with a relative molecular mass (M_r_) of about 60k and 30k, respectively.

## 2. Results

### 2.1. Characterization of TFF3 Forms in Human Bronchial Tissue Specimens

**Figure 1:**
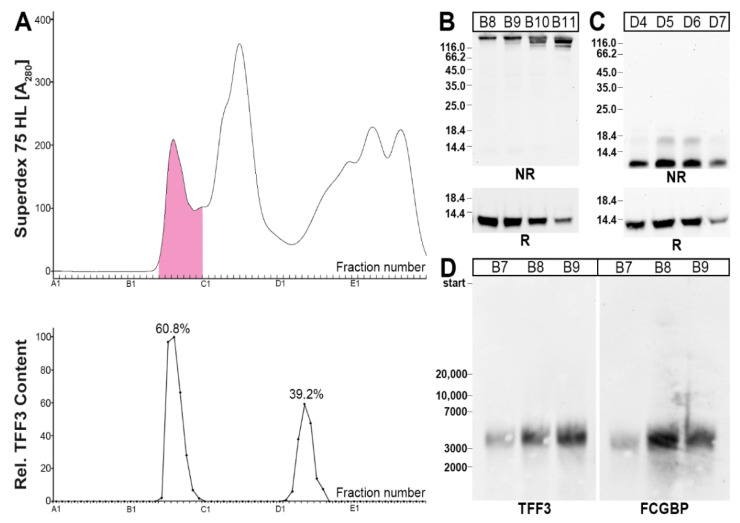
Analysis of a human bronchial tissue extract (L-13). (**A**) Elution profile after SEC on a Superdex 75 HL column as determined by absorbance at 280 nm (PAS-positive mucin fractions: pink). Underneath: Distribution of the relative TFF3 content as determined by Western blot analysis under reducing conditions and semi-quantitative analysis of the monomeric band intensities. (**B**) 15% SDS-PAGE under reducing (R) and non-reducing (NR) conditions, respectively, and subsequent Western blot analysis of the high-molecular-mass fractions B8–B11 concerning TFF3. (**C**) 15% SDS-PAGE under reducing (R) and non-reducing (NR) conditions, respectively, and subsequent Western blot analysis of the low-molecular-mass fractions D4–D7 concerning TFF3. (**D**) 1% agarose gel electrophoresis and subsequent Western blot analysis of high-molecular-mass fractions B7–B9 concerning TFF3 and FCGBP, respectively. Relative standard: DNA ladder (base pairs).

Human bronchial tissue extracts were separated by SEC and the TFF3 immunoreactivities tested (Figure 1). Clearly, the TFF3 content peaked in a high-molecular-mass region, which also contained periodic acid-Schiff (PAS)-positive mucins, and a low-molecular-mass region (Figure 1A). The high-molecular-mass form of TFF3 represents a disulfide-linked heterodimer with FCGBP (TFF3-FCGBP), as shown by Western blotting (Figure 1B,D). In contrast, the low-molecular-mass forms of TFF3 contain a band with an M_r_ of about 18k, which is probably a TFF3 homodimer [27,28], and monomeric TFF3, the latter of which is the predominant form (Figure 1C).

### 2.2. Characterization of Multiple TFF3 Forms in Human Bronchial Secretions

**Figure 2:**
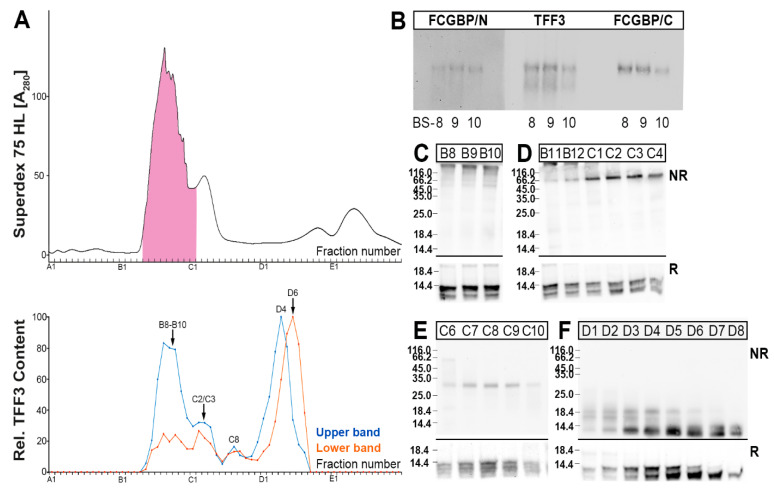
Analysis of a human bronchial secretion (BS-9). (**A**) Elution profile after SEC on a Superdex 75 HL column as determined by absorbance at 280 nm (PAS-positive mucin fractions: pink). Underneath: Distribution of the relative TFF3 content as determined by Western blot analysis under reducing conditions and semi-quantitative analysis of the monomeric double band: upper band intensities in blue; lower band intensities in red. (**B**) 1% agarose gel electrophoresis and subsequent Western blot analysis of high-molecular-mass fractions of three different bronchial secretions (BS-8, BS-9, BS-10). Analysis was with an antiserum against the *N*-terminal of FCGBP (FCGBP/N), the C-terminal of FCGBP (FCGBP/C), and TFF3, respectively. (**C**–**F**) 15% SDS-PAGE under non-reducing (NR) and reducing (R) conditions, respectively, and subsequent Western blot analysis concerning TFF3 of the fractions B8–B10 (**C**), B11–C4 (**D**), C6–C10 (**E**), and D1–D8 (**F**), respectively.

The separation of bronchial secretions via SEC and analysis of the TFF3 forms gave a different picture when compared with bronchial tissue extracts (Figure 2). Generally, under reducing conditions, TFF3 was always detectable as a double band, the upper band representing normal monomeric TFF3, and the lower band representing a shortened/degraded variant. A high-molecular-mass form of TFF3 peaking in fractions B8–B10 was detectable in a region which also contained PAS-positive mucins (Figure 2A). This form represents a disulfide-linked heterodimer with FCGBP as shown in Figure 2B using two different anti-FCGBP antisera. This has been shown for three different bronchial secretions (Figure 2B).

Furthermore, another disulfide-linked TFF3 entity was detectable mainly in fractions C2/C3, which appears with an M_r_ of about 60k (Figure 2D). Additionally, a disulfide-linked TFF3 form with a M_r_ of about 30k was detectable in fractions around C8 (Figure 2E). Finally, homodimeric TFF3 forms appeared in fractions D1–D5 (Figure 2F), and monomeric TFF3 forms appeared in fractions D2–D8 (Figure 2F). Of special note, each of the different TFF3 forms contains different amounts of the two TFF3 entities, i.e. the normal form (upper band; Figure 2A) and the degraded form (lower band; Figure 2A). For example, TFF3-FCGBP (e.g., B9) and homodimeric TFF3 (e.g., D4) mainly contain the normal TFF3 form (upper band in Figure 2A,C,F), whereas monomeric TFF3 (e.g., D6) mainly consists of the degraded form (lower band in Figure 2A,F).

**Figure 3:**
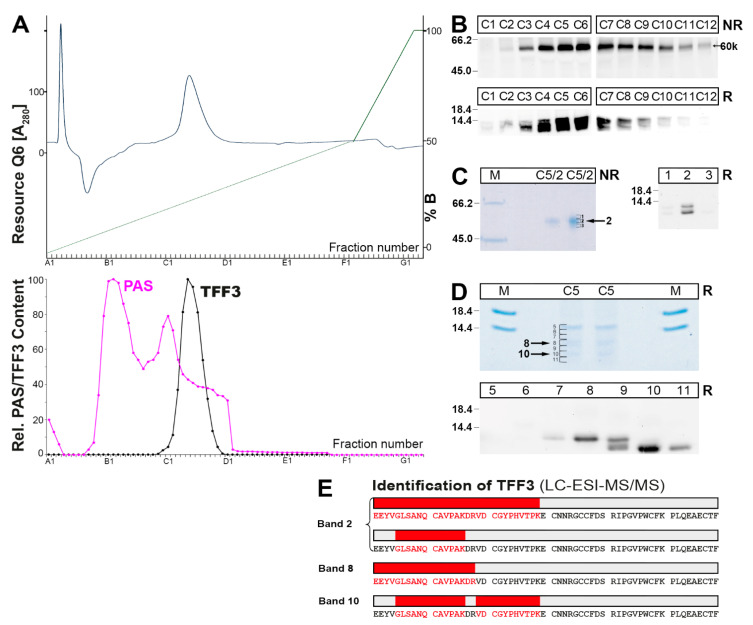
Purification of the 60k form of TFF3 from a human bronchial secretion (BS-9) and proteome analysis. (**A**) BS-9 was purified via SEC on a Superdex 75HL column (analogous to Figure 2) and fractions C1–C5 (60k form) were subjected to anion-exchange chromatography on a Resource Q6 column; elution profile after elution with a salt gradient (% B) as determined by absorbance at 280 nm. Underneath: Distribution of the relative TFF3 content as determined by Western blot analysis under reducing conditions and semi-quantitative analysis of the monomeric TFF3 intensities. The mucin content (PAS reaction) is shown in pink. (**B**) 15% SDS-PAGE under non-reducing (NR) and reducing (R) conditions, respectively, and subsequent Western blot analysis concerning TFF3 of the fractions C1–C12. Indicated is the 60k band under NR conditions. (**C**) 15% SDS-PAGE under NR conditions of fraction C5 (see Figure 3B), elution of the 60k-region (C5/2), and second separation of the excised band (C5/2) by 15% SDS-PAGE under NR conditions followed by Coomassie staining. Separation of the excised bands 1, 2, and 3 by 15% SDS-PAGE under reducing conditions and Western blot concerning TFF3. (**D**) 15% SDS-PAGE under reducing conditions of fraction C5 (see Figure 3B) followed by Coomassie staining. Separation of the excised bands 5–11 by 15% SDS-PAGE under reducing conditions and Western blotting concerning TFF3. (**E**) Results of the proteome analysis after tryptic in-gel digestion of the 60k-band (band 2 in Figure 3C), and bands 8 and 10 (Figure 3D), respectively. Identified tryptic peptides belonging to TFF3 are highlighted in red.

In order to prove that the 60k form really contains TFF3, this species was purified from a bronchial secretion via SEC, followed by anion-exchange chromatography (Figure 3). The 60k form accumulated in fractions C3–C10 (Figure 3A,B).

The presence of TFF3 in the 60k-band was further demonstrated by purification via preparative non-reducing SDS-PAGE, elution of the corresponding bands 1–3 and the identification of TFF3 by Western blot analysis under reducing conditions (Figure 3C). However, in band 2, two TFF3 forms were identified, i.e., the normal and a truncated form.

In addition, TFF3 was also directly released by reduction from the 60k-band, purified via preparative SDS-PAGE and elution (bands 5–11; Figure 3D), and each band was identified by Western blot analysis (Figure 3D). Again, two TFF3 forms were identified, i.e., a normal (band 8) and a truncated form (band 10); whereas band 9 contained both forms.

Furthermore, the purified bands with positive immunoreactivity for TFF3 (bands 2, 8, 10) were also analyzed by a proteomic approach after their tryptic digestion (Figure 3E). Clearly, the presence of TFF3 was demonstrated in all three bands. Band 2 contained both normal TFF3 as well as a truncated form; the latter lacked four amino acid residues (i.e., EEYV) at the N-terminus. Band 8 only contained the normal TFF3 form, whereas in band 10 only the *N*-terminally truncated form was detectable.

### 2.3. Characterization of Multiple TFF3 Forms in Human BAL Fluid

**Figure 4:**
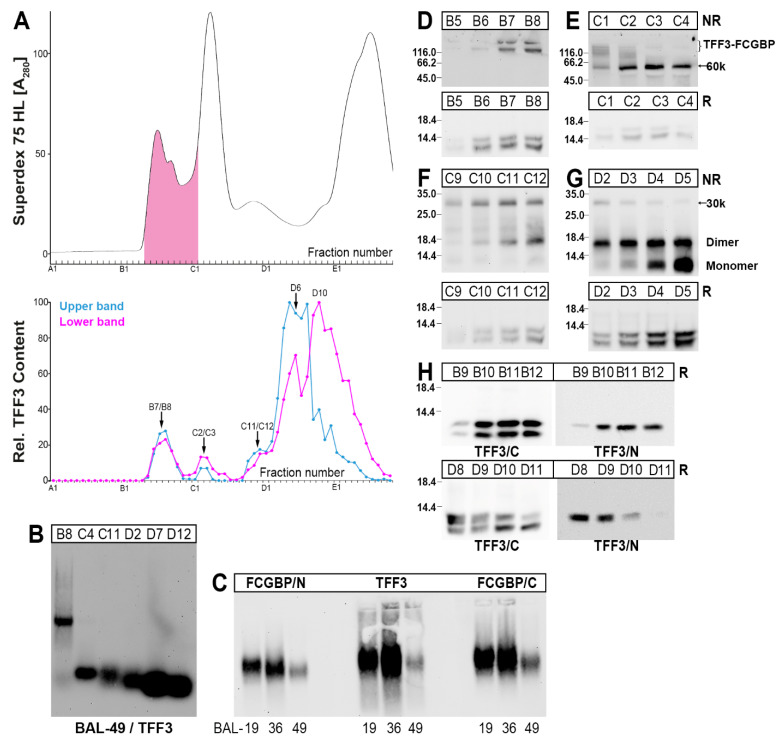
Analysis of a human BAL fluid (BAL-49). (**A**) Elution profile after SEC on a Superdex 75 HL column as determined by absorbance at 280 nm (PAS-positive mucin fractions: pink). Underneath: Distribution of the relative TFF3 content as determined by Western blot analysis under reducing conditions and semi-quantitative analysis of the monomeric double band: upper band intensities in blue; lower band intensities in red. (**B**) 1% agarose gel electrophoresis (AgGE) and subsequent Western blot analysis of high- (B8) and characteristic low-molecular-mass fractions (C4, C11, D2, D7, D12) concerning TFF3. (**C**) 1% AgGE and subsequent Western blot analysis of high-molecular-mass fractions of three different BAL fluids (BAL-19, BAL-36, BAL-49). Analysis was with an antiserum against the *N*-terminal of FCGBP (FCGBP/N), the *C*-terminal of FCGBP (FCGBP/C), and TFF3, respectively. (**D**–**G**) 15% SDS-PAGE under non-reducing (NR) and reducing (R) conditions, respectively, and subsequent Western blot analysis concerning TFF3 of the fractions B5–B8 (**D**), C1–C4 (**E**), C9–C12 (**F**), and D2–D5 (**G**), respectively. (**H**) 15% SDS-PAGE under reducing conditions of high- (B9–B12) and low-molecular-mass fractions (D8–D11) concerning TFF3. Analysis was with an antiserum against the C-terminal of TFF3 (TFF3/C) and the *N*-terminal of TFF3 (TFF3/N), respectively.

As a third source for the characterization of TFF3 forms in the human lung, BAL fluid was analyzed after separation by SEC (Figure 4).

A high-molecular-mass form of TFF3 peaking in fractions B7/B8 represents a heterodimer with FCGBP as shown by agarose gel electrophoresis (AgGE, Figure 4B). This has also been verified from specimens originating from three different individuals using two different anti-FCGBP antisera (Figure 4C). In contrast, all the other detectable forms were in the low-molecular-mass range (Figure 4B).

Heterodimeric TFF3 forms are also present in the 60k- (peak at C2/C3; Figure 4E) as well as the 30k-regions (peak at C11/C12; Figure 4F,G). In the low-molecular-mass region, it is likely that homodimeric and monomeric TFF3 forms are present (Figure 4G).

Under reducing conditions, TFF3 always appeared as a double band, i.e., an upper band representing normal monomeric TFF3, and a lower band representing a shortened/degraded form. The two TFF3 entities (normal versus shortened) appear differently in the different TFF3 forms (Figure 4A). In particular, homodimeric TFF3 mainly contains the normal TFF3 entity (Figure 4A); whereas monomeric TFF3 mainly consists of the shorted variant (Figure 4A,H).

By the use of two different anti-TFF3 antisera recognizing the *C*- and the *N*-terminal portions of TFF3, it could be demonstrated that the shortened TFF3 entity (lower band) contains a truncated *N*-terminal region (Figure 4H). This has been shown for both the low- and the high-molecular-mass forms, respectively (Figure 4H).

### 2.4. Interactions of TFF3-FCGBP with DMBT1gp^340^

**Figure 5:**
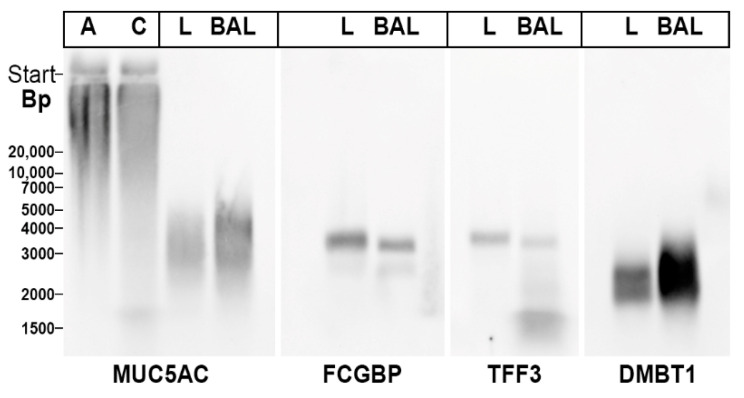
Analysis of a human bronchial tissue extract L-13 (L) and BAL-49 fluid (BAL). 1% agarose gel electrophoresis and subsequent Western blot analysis of high-molecular-mass fractions of L-13 and BAL-49 after SEC on Superdex 75 HL. Analysis was with antisera against MUC5AC, the C-terminal of FCGBP, TFF3, and DMBT1gp^340^, respectively. As a control for MUC5AC, extracts from human gastric antrum (A) and corpus (C), were analyzed.

In the past, homodimeric TFF3 was reported to interact in vitro with the agglutinin Deleted in Malignant Brain Tumor 1/gp340 (DMBT1gp^340^), a glycoprotein involved in mucosal innate immunity (review: [29]). Thus, we tested whether TFF3-FCGBP and DMBT1gp^340^ co-migrated after native AgGE possibly due to forming a complex (Figure 5). For comparison, the mucin MUC5AC was also analyzed.

TFF3-FCGBP was recognized as a band by both the anti-TFF3 and anti-FCGBP antisera. When compared with TFF-FCGBP, DMBT1gp^340^ appeared as a smear with a lower M_r_, whereas bronchial MUCAC showed a smear with a somewhat higher M_r_. This is an indication that in the bronchial tract TFF3-FCGBP and DMBT1gp^340^ are not associated. Furthermore, MUC5AC does not seem to form a complex with TFF3-FCGBP. Of special note, gastric MUC5AC appears with a much higher M_r_ than bronchial MUC5AC.

## 3. Discussion

In the course of these studies, different molecular forms of TFF3 were detected in the respiratory tract for the first time. In human bronchial tissue extracts, a high-molecular-mass form (heterodimeric TFF3-FCGBP) as well as low-molecular-mass forms (homodimeric and predominantly monomeric TFF3) were characterized (Figure 1). This is in agreement with results obtained from the human intestine [27] and saliva [28]. Generally, TFF3-FCGBP seems to be the predominant form of TFF3 in bronchial and intestinal tissues as well as saliva [27,28].

In contrast, in respiratory tract secretions (bronchial secretions and BAL fluid, respectively) additional TFF3 forms were detected, i.e., the heterodimeric 60k- and 30k-forms, respectively (Figure 2 and Figure 4). Additionally, little homodimeric TFF3 and mainly monomeric TFF3 represented the low-molecular-mass forms of TFF3 (Figure 2 and Figure 4). Furthermore, in the secretory specimens, TFF3 appeared as two entities, a normal TFF3 (upper band) and a degraded variant (lower band) lacking the four *N*-terminal amino acid residues (Figure 3 and Figure 4). Of note, and in contrast to the bronchial tissue extracts, in the secretory material the TFF3-FCGBP heterodimer does not represent the predominant TFF3 form anymore (Figure 2 and Figure 4).

The results were obtained from specimens from patients with a COPD background, which is characterized by an abnormal inflammatory reaction. This might be a limit of this study as both TFF3 and FCGBP are up-regulated during inflammation, e.g., by interleukin-13 (IL-13). However, both TFF3 and FCGBP are also typically expressed in the mucous cells of submucosal glands of normal control lung tissue [12,30]. Thus, the presence of the TFF3-FCGBP heterodimers in healthy lung tissue can be anticipated.

### 3.1. TFF3 from the Human Respiratory Tract Forms High-Molecular-Mass Heterodimers with FCGBP

**Figure 6:**
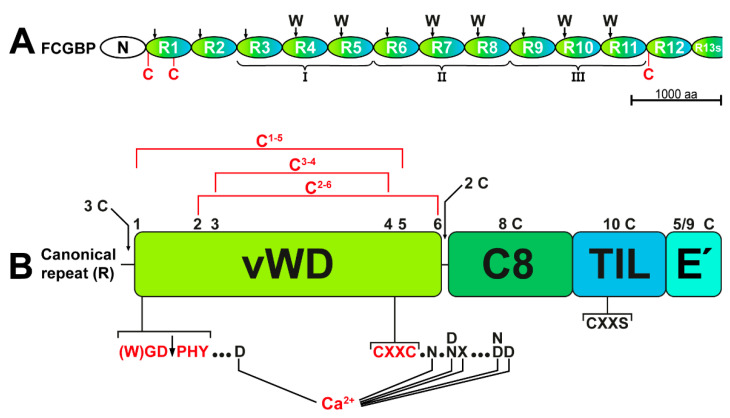
Schematic structures of FCGBP (**A**) and the canonical repeat with similarity to D assembly domains (**B**), respectively. (**A**) The autocatalytic cleavage sites in FCGBP (R1–R11) are indicated by arrows, W indicating preferred cleavage sites [27]. The repeats R3–R5, R6–R8, and R9–R11 are parts of longer repeats (I, II, III). Additional cysteine residues (C) in R1 and R12 are shown in red (for details, see [27]). Scale bar: 1000 amino acid residues. (**B**) Depicted is a canonical repeat (R) consisting mainly of cysteine-rich vWD, C8, TIL, and E’ modules (for details, see [27]). The number of cysteine residues is given above each module including additional cysteine residues *N*-terminal to vWD (3 C) and between vWD and C8 (2 C), respectively. In the vWD module, the six conserved cysteine residues are numbered and the disulfide bridges are indicated as deduced from vWF [47]. Also shown are the six conserved coordination sites for Ca^2+^ within the vWD modules as deduced from vWF [47]. Outlined also are the conserved CXXC and CXXS motifs.

Currently, the biological role of TFF3-FCGBP and FCGBP, respectively, is not known. FCGBP is a cysteine-rich glycoprotein with an estimated M_r_ of about 650k at least (the precursor comprises of 5405 amino acid residues; NCBI reference sequence: NP_003881.2; [46]) containing an *N*-terminal domain (FCGBP-N) followed by 12.5 repeats (R1–R12, and the shortened repeat R13s) arranged in tandem with similarity to D assembly domains (Figure 6A; for details, see [27]). The latter were originally detected in von vWF and later on in different gel-forming mucins from *Xenopus laevis* (frog integumentary mucin FIM-B.1) to human (MUC2, MUC5AC, MUC5B, MUC6) [33,35,47]. During multimerization of both vWF and the gel-forming mucins, an inter-molecular disulfide linkage is formed between D3 assemblies [35,47]. D3 dimerization of vWF requires Ca^2+^, and Ca^2+^–binding sites were identified in most of its vWD domains [47]. These coordination sites for Ca^2+^ are conserved in vWD domains in both gel-forming mucins [34] and FCGBP (Figure 6B). As a hallmark, D assemblies contain 4 cysteine-rich modules, i.e., von Willebrand D domain (vWD, eight cysteines), C8 (eight cysteines), TIL (trypsin inhibitor-like, 10 cysteines), and E (6 cysteines) [47]. These four modules pack in a highly circular fashion against one another in each D assembly and the vast majority of disulfide bridges are within a single cysteine-rich module [47].

In FCGBP, each of the repeats R1–R12 contains assembly domains built up of somewhat modified versions of vWD (mostly six cysteines), C8, TIL, and E modules (Figure 6); whereas R13s consists of a vWD domain only (for details, see [27]). By analogy with vWF [47], vWD domains in FCGBP are expected to be stabilized by three conserved disulfide bridges in the order C^1-5^, C^2-6^, and C^3-4^ (Figure 6B). Furthermore, all 13 vWD modules contain a conserved **C**^4^GL/A**C**^5^G motif [27,46], known as a CXXC motif (see Figure 6B), which is essential for the catalysis of redox reactions in thiol:disulfide oxidoreductases [48,49]. The conserved **C**GL**C**G motif is also present in porcine submaxillary mucin and MUC5AC, where it plays a role for *N*-terminal multimerization [43]. In addition, all TIL domains (10 cysteine residues) in FCGBP possess a conserved C^3^XXS motif (Figure 6B; for details, see [27]), which is missing in vWF, but is a characteristic constituent of many disulfide isomerases, such as AGR2 [50], and the disulfide catalyst quiescin sulfhydryl oxidase 1 (QSOX1) [51]. Taken together, these are indications that FCGBP might be involved in disulfide isomerization reactions and covalent disulfide crosslinking.

As a hallmark, 11 of the 13 vWD domains (R1 to R11) contain the motif (W)GD↓PHY (Figure 6A), which is subject to the autocatalytic cleavage between D and P (Figure 6B), the preceding W residue accelerating the cleavage reaction [27]. Cleavage of this motif was first documented for rat Fcgbp in 2002 [52]. Remarkably, this motif is lacking in vWF. Similar cleavages of WGD↓PHY motifs occur in the vWD4 domains of the mucins MUC2 and MUC5AC [53,54] as well as in a variety of other proteins, such as toxin proteins from Gram-negative pathogens [55] and repulsive guidance molecules [56]. Cleavage can occur late in the secretory pathway, preferentially at a pH below 6 [53], and is probably Ca^2+^ dependent [55]. This is the typical milieu of mucin storage granules, which is characterized by increased [H^+^] and [Ca^2+^] [57]. The pH dependence might explain why the processing of FCGBP is changed in prostate secretion of *Atp12a*-deficient mice, where the acidification of prostate fluid is disturbed [58]. Of note, after proteolytic cleavage, the FCGBP fragments are still cross-linked by disulfide bridges under non-reducing conditions [27,42,46,52], probably due to the disulfide bridge C^1-5^ in the vWD modules (Figure 6B). C^1^ is in close proximity to the (W)GD↓PHY cleavage site and C^5^ is part of the CXXC^5^ motif (Figure 6B). This might be a sign that the disulfide bridge C^1-5^ becomes shear-sensitive particularly after proteolytic cleavage, when it changes from an intra- to an inter-chain bridge. Furthermore, the conserved six coordination sites for Ca^2+^ are also located in close proximity to both the (W)GD↓PHY motif (one coordination site) and the CXXC motif (five coordination sites, Figure 6B). Taken together, this is a strong indication for a Ca^2+^ dependent cleavage followed by a conformational change, which would allow the *N*-terminally generated proline residue to be deeply buried in the protein core as shown for a repulsive guidance molecule [56]. Furthermore, the β-carboxyl group of the *C*-terminal aspartate generated after the cleavage could be covalently linked to the ε-amino group of an internal lysine by nucleophilic attack [55]. Here, very stable isopeptide bonds could create crosslinks, either within the same molecule or neighboring FCGBP molecules. However, FCGBP does not crosslink to mucins, as claimed previously [42].

In vWF, the D1 and D2 assemblies are a prerequisite for intracellular packing of the precursor in Weibel-Palade bodies and the D’D3 assembly is required for multimerization of vWF via linkage of two free cysteines (C-1099, C-1142) [47]. These interactions require Ca^2+^ and low pH [47]. In order to prevent premature inter-molecular disulfide formation, the two free cysteine residues are not surface exposed [47]. FCGBP contains 435 cysteine residues and this odd number suggests the presence of at least one free thiol, allowing the formation of an inter-molecular disulfide bridge [27]. Based on a comparison of the 13 repeats in FCGBP [27], three cysteine residues might be candidates for free cysteines (Figure 6A), i.e., the additional C-593 in R1 (in vWD1), the additional C-4853 in R12 (just before vWD12), and one of the three *N*-terminal cysteines (C-449, C-455, C-464) in R1 (just before vWD1). Theoretically, R13 could also be involved in the formation of disulfide bridges [27]. The three proposed cysteine residues in R1 and R12 (Figure 6A) could be particularly involved in the formation of inter-molecular disulfide bridges with TFF3 and with another FCGBP molecule, respectively. This would allow the formation of a variety of different FCGBP dimers and oligomers.

### 3.2. TFF3-FCGBP Forms Oligomers and Does Not Bind to DMBT1gp^340^

The M_r_ of TFF3-FCGBP can be roughly estimated by extrapolation after separation by non-denaturing AgGE and the use of commercial protein markers (see Section 4.3). Typically, the observed M_r_ is far more than 300k [27] and a graphical extrapolation revealed values of about 6–8 × 10^6^. This is a clear indication that TFF3-FCGBP forms oligomers, which may consist of about 10 monomeric units. The observed formation of TFF3-FCGBP oligomers is in agreement with a recent report [59].

TFF3 with its single free C-terminal cysteine residue would be ideally suited to controlling FCGBP oligomerization by acting as a free thiol scavenger for FCGBP; this could limit the size of the FCGBP oligomers and also influence their structure (linear, circular, branched). In addition, the expected lectin activity of TFF3 could stabilize the TFF3-FCGBP oligomers; the latter would be an ideal extracellular matrix playing a key role in the innate immune defense of mucous epithelia, in addition to the network of gel-forming mucins. Such a mechanism would require the expression of both FCGBP and TFF3 in about equal molar amounts. This has been demonstrated in the past [27]. Protection by TFF3-FCGBP is of particular importance during inflammatory processes. In agreement with this, both TFF3 and FCGBP are upregulated by interleukin-13 (IL-13) [60,61,62].

DMBT1gp^340^ is a glycoprotein typically expressed in many mucous epithelia including the submucosal glands of the bronchi, where it plays an important role in innate immune defense [63]. For example, it binds to lipopolysaccharides and various microorganisms (bacteria as well as viruses), and also interacts with surfactant proteins [63]. Of special note, DMBT1gp^340^ was described as a TFF3 binding protein in vitro [64]. However, the results presented in Figure 5 argue against the association of TFF3-FCGBP with DMBT1gp^340^ in bronchial extracts as well as BAL fluid. In addition, the mucin MUC5AC also does not seem to be associated with TFF3-FCGBP in the respiratory tract (Figure 5). The latter would be in line with a recent report excluding the covalent linkage of FCGBP and the intestinal mucin MUC2 [42]. Taken together, TFF3-FCGBP seems to be an independent high-molecular-mass constituent of the respiratory mucus, probably with a role in innate immunity.

### 3.3. Respiratory Tract Secretory Specimens Contain 60k and 30k TFF3 Heterodimers

In bronchial secretions (Figure 2D) as well as in BAL fluid (Figure 4E), a TFF3 immunoreactive 60k-band appeared after SDS-PAGE under non-reducing conditions which released monomeric TFF3 forms after reduction (Figure 3D). After purification of the 60k-band from a bronchial secretion (Figure 3), TFF3 could be identified by proteome analysis directly in the 60k-band (Figure 3E) as well as after its reductive release (Figure 3E). Thus, it can be concluded that TFF3 is disulfide-linked to a partner protein with an M_r_ of about 53k. Unfortunately, it was not possible to identify the partner protein unambiguously. It should be mentioned that a similar 60k-band was recently identified in the human stomach representing a disulfide-linked heterodimeric form of TFF1 [36], the latter having high similarity with TFF3 [4]. However, identification of the partner protein is also still missing [36]. Theoretically, one could propose that the 60k heterodimeric form of TFF3 might be a degradation product of TFF3-FCGBP. However, we could not find indications supporting this hypothesis. Nevertheless, and remarkably, in the course of characterizing the 60k-band, multiple tryptic fragments of the mucins MUC5AC and MUC5B were identified, all clustering in the *N*-terminal regions of these mucins (MUC5AC: within the 685 *N*-terminal amino acid residues of the precursor; MUC5B: within the 676 *N*-terminal amino acid residues of the precursor; data not shown). Currently, it cannot be excluded that in bronchial secretions, minute amounts of TFF3 might form disulfide-linked heterodimers with *N*-terminal fragments of MUC5AC and MUC5B. However, these proteomic results are an indication that in bronchial secretions both MUCAC and MUC5B are proteolytically cleaved at least at the *N*-terminal. The observed degradation is in line with the results from Figure 5, where respiratory MUC5AC has a much lower M_r_ than gastric MUC5AC.

Furthermore, in bronchial secretions (Figure 2E), as well as in BAL fluid (Figure 4F) minute amounts of a TFF3 form with a M_r_ of about 30k was detectable. This is expected to represent a heterodimeric TFF3 form with a disulfide-linked partner protein with an estimated M_r_ of about 23k. Attempts to identify the partner protein failed.

### 3.4. Degradation of TFF3 in Bronchial Secretions

One of the hallmarks of the respiratory secretions (bronchial secretions and BAL fluid, respectively) is the occurrence of two TFF3 entities after reducing SDS-PAGE (Figure 2 and Figure 4). Here, in addition to the normal form, a shortened TFF3 form appeared, the latter missing the *N*-terminal four amino acid residues EEYV, as shown for a bronchial secretion by proteome analysis (Figure 3E). Also, BAL fluid contains an *N*-terminally shortened TFF3 entity (Figure 4H). In contrast, in bronchial tissue extracts the shortened TFF3 form is missing.

A proteolytic degradation seems to be the most reasonable explanation for generating the shortened TFF3-form. However, this is rather unusual for TFF peptides, as the cysteine-rich TFF domains are well known for their resistance to proteolytic degradation [2]. Currently, the nature of this enzymatic process is not clarified. One potential candidate would be the neutrophil elastase, which is a serine protease released from neutrophils, the latter representing the typical inflammatory cells in the lung [65,66]. Generally, proteases play a major role in chronic lung diseases [67]. Neutrophil elastase has a relatively broad substrate specificity and preferentially cleaves after valine, cysteine, alanine, methionine and isoleucine residues (P1 position) [68]. Thus, the observed cleavage after Val-4 (Figure 3E) would be in agreement with the cleavage of TFF3 by the neutrophil elastase. Of note, in human bronchial secretion, TFF3 seems to be protease resistant in the TFF3-FCGBP heterodimer as well as the homo-dimeric form, whereas monomeric TFF3 predominantly appears in the truncated form (Figure 2A). Thus, dimerization seems to protect TFF3 from proteolytic cleavage, perhaps by masking the potential cleavage site. The lack of proteolytic degradation in the tissue specimens (Figure 1) might be due to the lack of inflammation in these samples.

Currently, there are no convincing data defining unambiguously the biological role(s) of monomeric and homo-dimeric TFF3 or the shortened TFF3. Generally, TFF3 (and probably also fragments thereof) do not seem to support the restitution of mucous epithelia significantly as their motogenic and anti-apoptotic effects are rather weak [3], and could result from degradation of the TFF3-FCGBP heterodimer or from being a side product of incomplete heterodimer formation. Based on the various reports on the anti-apoptotic effects of TFF3 [3,69], one might speculate that TFF3 protects alveolar type 2 (AT2) cells from apoptosis. This could be due to a lectin-triggered receptor blocking/activation [4,62] by TFF3 and could have medical significance, as AT2 cells not only orchestrate pulmonary innate immunity and secrete a surfactant, but also act as progenitor cells for the alveolar epithelium [70]. Such a mechanism might be of clinical importance, particularly in the development of COPD [70]. However, TFF3 was also reported to increase apoptosis (pro-apoptotic effect [71]). Furthermore, monomeric TFF3 with its free thiol could act as a protective scavenger for ROS similar to that proposed for TFF1 [36,37,72]. By analogy with reports concerning the cervical mucus, TFF3 might even also affect the rheological properties of the respiratory mucus [19].

## 4. Materials and Methods

### 4.1. Human Specimens

All investigations followed the declaration of Helsinki and were approved by the Ethics Committee of the Medical Faculty of the Otto-von-Guericke University Magdeburg (codes: 09/98 February 1998, 102/03 July 2003, 53/05 July 2005, 82/11 July 2011). All patients gave written informed consent. Here, representative results are presented obtained with specimens from the lungs from four patients (L-13, L-14, L-15, L-19), bronchial secretions (BS) from three patients (BS-8, BS-9, BS-10), and also BAL fluids from three patients (BAL-19, BAL-36, BAL-49). All patient-related procedures—bronchoscopies and surgical resections—were performed with a clinical indication, i.e., peripheral lung cancer (T1 to T2). All patients were smokers or former smokers and suffered from concomitant COPD GOLD Stages I to III. Patients were included when a visible mucous hypersecretion as a sign of chronic bronchitis was present. Patients with predominant emphysema without bronchial hypersecretion were excluded.

Lung tissue was obtained from resected specimens from patients undergoing thoracic surgery because of peripheral lung cancer. Specimens were included in the study only, when they were free from malignancy, and not used for pathological workup.

BS and BAL were obtained during flexible bronchoscopy under local anesthesia. BS were collected by aspiration via the working channel of the bronchoscope into a special secretion trap. BAL was performed in the right middle lobe by rinsing and aspiring with five aliquots of 20 mL sterile saline.

### 4.2. Protein Purification by SEC and Anion-Exchange Chromatography

The extraction of lung specimens was performed with a five-fold amount (*w*/*v*) of buffer (30 mM NaCl, 20 mM Tris-HCl pH 7.0 plus protease inhibitors (0.5 mM benzamidine hydrochloride, 0.1 mM Pefabloc SC, 1 µg/mL leupeptin)) in a Precyllys^®^24 lyser/homogenizer as described previously [73,74].

Extracts or diluted BS or BAL fluid (6–8 mL) were fractionated by SEC with the ÄKTA^TM^ FPLC system (Amersham Biosciences, Freiburg, Germany) as previously reported (fraction numbering: A1–A12, B1–B12, etc.) [75] using a HiLoad 16/600 Superdex 75 prep grade (S75HL; 20 mM Tris-HCl pH7.0, 30 mM NaCl plus protease inhibitors; flow rate: 1.0 mL/min; 2.0 mL fractions) column as described [36]. Additionally, anion-exchange chromatography was undertaken using a Resource Q6 column (salt gradient from 20 mM Tris-HCl pH7.0. (buffer A) to 20 mM Tris-HCl pH7.0 + 1 M NaCl (buffer B); flow rate: 4.0 mL/min, 1.0 mL fractions) was applied for further purification as reported [36,76].

### 4.3. SDS-PAGE, Agarose Gel Electrophoresis, and Western Blot Analysis

**Figure 7:**
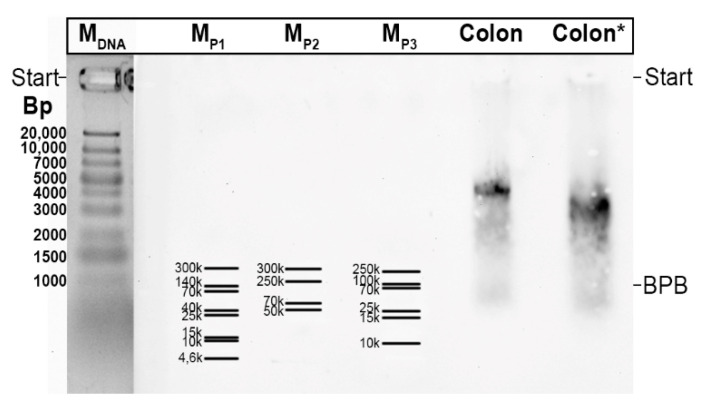
1% agarose gel electrophoresis (AgGE) and subsequent Western blotting. Shown is a comparison of the DNA ladder (Thermo Fisher Scientific; base pairs; M_DNA_) with different commercial, pre-stained protein markers: ProSieve QuadColor (00193838, Lonza, Rockland, ME, USA; M_P1_), Spectra (26625, Thermo Fisher Scientific, M_P2_), and Protein Ladder (26619, Thermo Fisher Scientific; M_P3_). The relative DNA standard is indicated in base pairs (bp); whereas for the protein markers the M_r_ is given. For comparison, TFF-FCGBP was detected in a high-molecular-mass fraction of a human colon extract by the use of an anti-TFF3 antiserum. The sample indicated by star (Colon*) was boiled before AgGE. BPB, bromophenol blue.

All methods were described in previous publications in detail, i.e., denaturing SDS-PAGE under reducing or non-reducing conditions, protein staining with Bio-Safe Coomassie Stain G-250 without fixation, non-denaturing AgGE, and periodic acid-Schiff (PAS) staining of mucins (dot blot) [27,75,77]. As a relative standard for non-denaturing AgGE, the GeneRuler 1 kb Plus DNA Ladder (Thermo Fisher Scientific Baltics UAB, Vilnius, Lithuania) was used, as most commercial protein markers do not cover the M_r_ range above 300k, which is typical of TFF3-FCGBP (Figure 7). Staining of the DNA ladder was done with GelRed^®^ (41011, Biotium, Fremont, CA, USA).

Western blot analysis after SDS-PAGE or AgGE was performed as reported [74,75,77,78]. Gels after non-reducing SDS-PAGE were subjected to post-in-gel reduction before blotting, as published previously [75].

For immune-detection, polyclonal rabbit antisera were mainly used. TFF3 was detected with anti-hTFF3-8 against the C-terminal peptide FKPLQEAECTF of human TFF3 [28]. For specific purposes (see Figure 4H), the commercial monoclonal mouse antibody TFF3-15C6 (nanoTools Antikörpertechnik GmbH & Co. KG., Teningen, Germany) against amino acids 1–12 of human TFF3 (termed TFF3/N) was applied. FCGBP was generally detected with PAP389Hu01 (Cloud-Clone Corp., Katy, TX, USA) against amino acids 5176 -5344 of human FCGBP (termed FCGBP/C). For specific purposes (see Figure 2 and Figure 4), HPA003564 (Sigma-Aldrich Chemie, Taufkirchen, Germany) against amino acids 289–417 of human FCGBP (termed FCGBP/N) was used. DMBT1gp^340^ was detected with the monoclonal antibody HYB 213-6 kindly provided by Prof. U. Holmskov (University of Southern Denmark, Odense, Denmark) [63]. Detection of the mucin MUC5AC was with the polyclonal antiserum anti-hMUC5AC-2 raised against the peptide RNQDQQGPFKMC; the latter is localized within multiple CysD domains, which characteristically interrupt the Pro-Thr-Ser-rich O-glycosylated domain [79]. 

### 4.4. Identification of Proteins by Bottom-Up Proteomics

For protein identification, gel bands were subjected to tryptic digestion, followed by liquid chromatography coupled to electrospray ionization and tandem mass spectrometry (LC-ESI-MS/MS), and resulting data were processed and analyzed with a search engine as described [76].

#### 4.4.1. Tryptic In-Gel Digestion

Proteins in gel bands were digested according to Shevchenko et al. [80]. Briefly, 100% acetonitrile (ACN) and 100 mM NH_4_HCO_3_ were used for shrinking and swelling, respectively. Disulfide bonds of the proteins in the gel-band were reduced with 10 mM dithiothreitol in a 100 mM NH_4_HCO_3_ buffer. Alkylation of the SH-groups of cysteines was undertaken with 55 mM iodacetamide dissolved in 100 mM NH_4_HCO_3_. Tryptic digestion of the proteins in the gel bands were performed at 37 °C for 16 h in a NH_4_HCO_3_ buffer (50 mM) containing 10% ACN and 8 ng/µL trypsin (sequencing-grade). The tryptic peptides were transferred into the supernatant with 2% formic acid (FA), 80% ACN. The solvent of the supernatant was removed by evaporation in a vacuum centrifuge. Prior to the LC-MS/MS analysis, the dried peptides were dissolved in 20 µL 0.1% FA (sample application buffer, solvent A).

#### 4.4.2. LC-MS/MS Analysis of the Tryptic Peptides

Tryptic peptides dissolved in the sample application buffer (0.1% FA in HPLC grade water, solvent A) were injected (5 µL) into a nano-liquid chromatography system (Dionex UltiMate 3000 RSLCnano, Thermo Fisher Scientific, Bremen, Germany) equipped with a trapping column (Acclaim PepMap µ-precolumn, C18, 300 µm × 5 mm, 5 µm, 100 Ǻ, Thermo Fisher Scientific, Bremen, Germany), a separation column (Acclaim PepMap 100, C18, 75 μm × 250 mm, 2 µm, 100 Ǻ, Thermo Fisher Scientific, Bremen, Germany) and connected with an electrospray-ionization (ESI) source (fused-silica emitter: I.D. 10 μm; New Objective, Woburn, MA, USA; capillary voltage of 1650 V) to a trybrid mass spectrometer (MS) comprising a quadrupole, a linear trap and an orbitrap (Orbitrap Fusion, Thermo Fisher Scientific, Bremen, Germany). After washing the tryptic peptides for 5 min with 2% solvent B (0.1% FA in ACN) with 5 μL/min, they were separated using a flow rate of 200 nL/min with a gradient from 2% to 30% B in 30 min. The positive ion mode and data dependent acquisition mode (DDA) was used for mass spectrometry. Every second, over a *m*/*z* range from 400–1500 (resolution of 120,000 FWHM at m/z 200; transient length: 256 ms; maximum injection time: 50 ms; AGC target: 2 × 10^5^), a MS scan was performed. For fragmentation, an HCD collision energy of 28%, an intensity threshold of 2 × 10^5^ and an isolation width of 1.6 *m*/*z* was chosen. In the ion trap, MS/MS spectra were measured (scan-rate: 66 kDa/s; maximum injection time: 200 ms; AGC target: 1 × 10^4^; underfill ratio of 10%; isolation width of 2 *m*/*z*).

#### 4.4.3. LC-MS/MS Data Processing and Protein Identification

LC-MS/MS data were processed with the software Proteome Discoverer 2.4.1.15 (Thermo Fisher Scientific, Bremen, Germany). Proteins were identified by using the search engine Sequest HT and the protein database SwissProt (www.uniprot.org, 2016). The following search parameters were used: Species: homo sapiens, precursor mass tolerance: 10 ppm; fragment mass tolerance: 0.2 Da. Missed cleavages: two were allowed; fixed modification: carbamidomethylation on cysteine residues; variable modification: oxidation of methionine residues. FDR: 1% using Percolator. For a reliable identification, at least two unique peptides per protein were used.

## 5. Conclusions and Medical Perspectives

Taken together, the oligomers of TFF3-FCGBP are the predominant TFF3 form in the human respiratory tract. TFF3 might play a role in the oligomerization of FCGBP. TFF3-FCGBP is probably a key component of the disulfide-linked extracellular matrix with a major role in mucosal innate immune defense. For example, FCGBP could influence the adherence of microorganisms as well as their clearing [3]. FCGBP has even been proposed to act as a trap for viral-antibody complexes [81]. It could also influence the metastasis of tumors; however, the observations are contrary [40]. The application of TFF3, TFF3-FCGBP, FCGBP or FCGBP fragments, e.g. during BAL, or a drug-mediated modulation of FCGBP expression might be novel strategies to support the innate immune defense of the respiratory tract (but also of other mucous epithelia) against microbial infections including viruses, such as SARS-CoV-2.

## Abbreviations

AgGE	Agarose gel electrophoresis
BAL	Bronchoalveolar lavage
BS	Bronchial secretion
COPD	Chronic obstructive pulmonary disease
FCGBP	IgG Fc binding protein
PAS	Periodic acid Schiff
ROS	Reactive oxygen species
TFF	Trefoil factor family
vWF	von Willebrand factor
